# Spinal subdural hematoma with sudden onset of paraplegia in a patient on oral anticoagulant therapy

**DOI:** 10.1002/ccr3.8895

**Published:** 2024-05-15

**Authors:** Hayato Ito, Hirohito Hirata, Tomohito Yoshihara, Masatsugu Tsukamoto, Kazuyuki Watanabe, Yoshiaki Egashira, Masaaki Mawatari, Tadatsugu Morimoto

**Affiliations:** ^1^ Department of Orthopedic Surgery, Faculty of Medicine Saga University Saga Japan; ^2^ Department of Orthopaedic Surgery Fukushima Medical University Fukushima Japan; ^3^ Department of Radiology, Faculty of Medicine Saga University Saga Japan

**Keywords:** anticoagulant therapy, lower back pain, paralysis of both lower limb, spinal subdural hematoma

## Abstract

Spinal subdural hematoma is uncommon but may become more prevalent with increasing anticoagulant use. Early diagnosis from symptoms like lower back pain and leg paralysis is crucial for timely intervention.

## 1 INTRODUCTION

Spinal subdural hematoma (SSDH) is a rare disease. However, once SSDH occurs, it results in a critical spinal cord injury. SSDH usually happens generally in patients with risk factors being anticoagulation and coagulopathy resulting from hematological disease.^1^, ^2^, ^3^, ^4^, ^5^

In recent years, the number of patients taking oral anticoagulants has increased.^6^ In the case of acute subdural hematoma (SDH) regarding intracranial region, the reduction of coagulation ability due to anticoagulant drugs can prolong the severity of damage and bleeding time. However, if appropriate coagulation ability can be restored with reversal agents, it has been reported that there is no impact on the prognosis.^7^ Clinicians who use anticoagulant therapy, as well as spinal surgeons, need to be more knowledgeable about SSDH in relation to anticoagulant therapy, because early diagnosis and treatment have prognostic relevance.^1^, ^2^, ^3^, ^4^, ^5^ We experienced a case of SSDH with paralysis of both lower limbs associated with anticoagulant medication. We will discuss the medical history and clinical characteristics, imaging findings, and prognosis in relation to SSDH, referring to the relevant literature.

## 2 CASE HISTORY AND EXAMINATION

A 76‐year‐old male was emergently transported to our hospital with lower back pain and paralysis of both lower limbs. He had undergone coronary artery bypass surgery for angina pectoris 1 year before he was transported to our hospital and was taking aspirin and warfarin.

On the day, around 3 a.m. he developed lower back pain and paralysis in both legs. He was rushed to a nearby hospital, where X‐rays and CT scans revealed no obvious findings, and the patient was referred as a spinal cord infarction was suspected.

**FIGURE 1:**
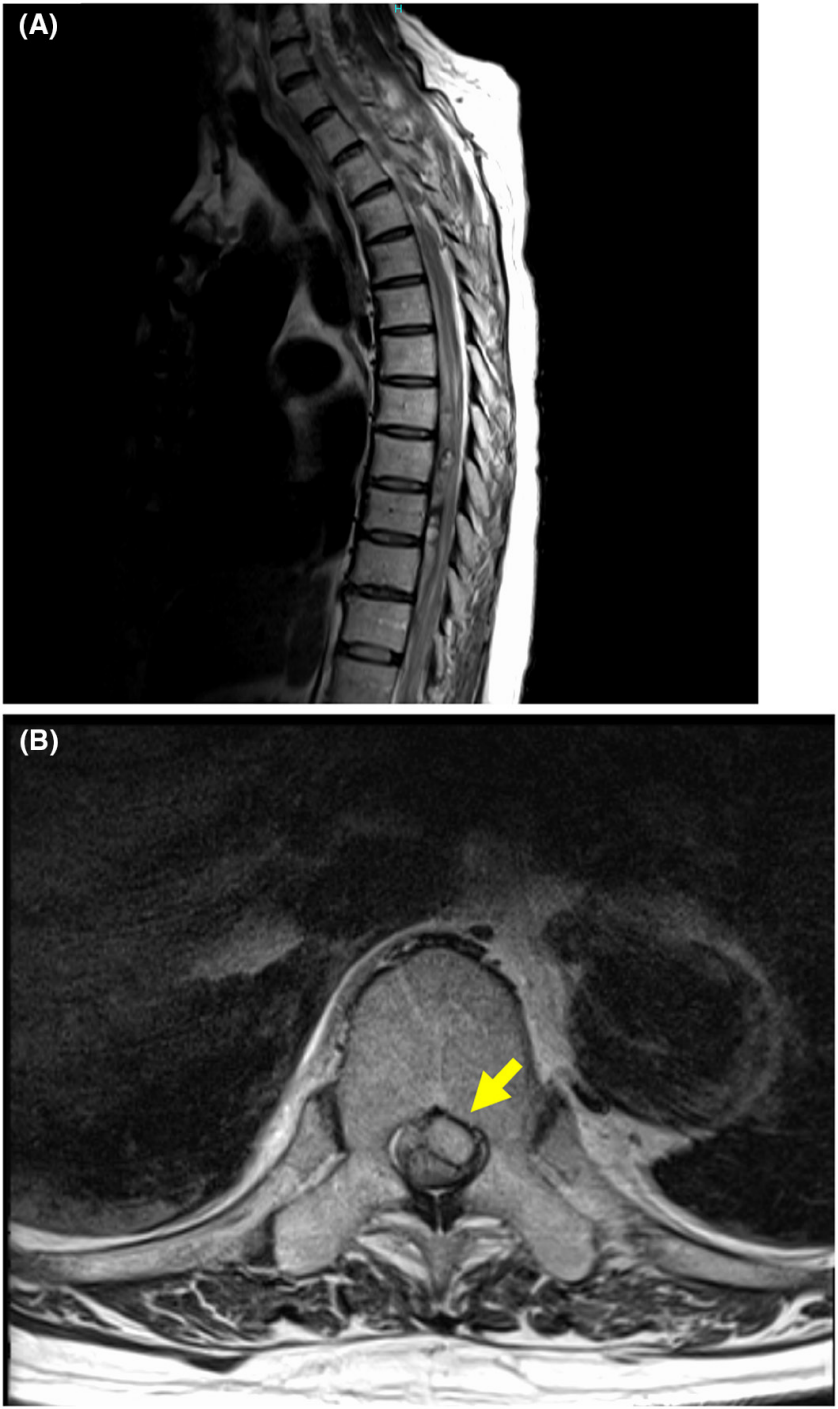
Preoperative MRI. (A) Sagittal T2‐weighted MRI revealed evidence of intradural hematoma from the T7 level to the T10 level. (B) Axial T2‐weighted MRI revealed a hematoma on the ventral side of the spinal cord at the T10 level.

He was emergently transported to our hospital around 7 a.m. the same day. He had lost sensation below the navel, and the manual muscle test for both lower limbs was reduced to 0–1, with bladder and rectal disturbances noted. Suspecting thoracic spinal cord damage, magnetic resonance imaging (MRI) was performed. The MRI revealed a SDH anterior to the thoracic spinal cord from levels T7 to T10, and changes in spinal cord signal intensity suggestive of edema due to compression from level T2 to T10. MRI findings showed no findings suggestive of a spinal dural arteriovenous fistula (AVF). The patient was diagnosed with a SDH from T7 to T10 (Figure 1A,B). Blood tests at the time of admission showed an increased PT‐INR of 1.94 (normal range 0.9–1.1) and a reduced platelet count of 95,000/μL (normal range 131,000–362,000/μL).

### 2.1 Differential diagnosis

Neoplastic lesions were considered as a differential diagnosis for the bilateral lower limb paralysis. These were excluded because a previous CT scan after symptom onset did not reveal any occupying lesions in the spinal canal. Additionally, the possibility of an abscess was considered based on the MRI images, but we considered it unlikely given the absence of a fever and a WBC of 7500, which was not elevated. Although the PT‐INR was prolonged, it was good as a control. After surgery, oral aspirin and warfarin were discontinued. Informed consent was obtained for emergency surgical treatment for the patient.

### 2.2 Treatment

Vitamin K is used preoperatively to reverse warfarin's anticoagulant action. Emergency surgical treatment was performed at 4 p.m. the same day.

A laminectomy was carried out from T6 to T10, and upon opening the dorsal dura mater, it was found to be filled with black hematoma. The spinal arachnoid membrane was adherent, and the hematoma surrounding the spinal cord was removed. Despite a comprehensive search, the source of the hemorrhage remained unidentified. There were no obvious signs of vascular dilation, and a spinal dural AVF was not observed.

After removing the hematoma, the dura mater was closed using the watertight suture technique and tissue repair agents. The possibility of spinal cord edema was considered, but an artificial dura mater patch was not used because there was ample space around the spinal cord after the removal of the hematoma.

### 2.3 Outcome and follow‐up

**FIGURE 2:**
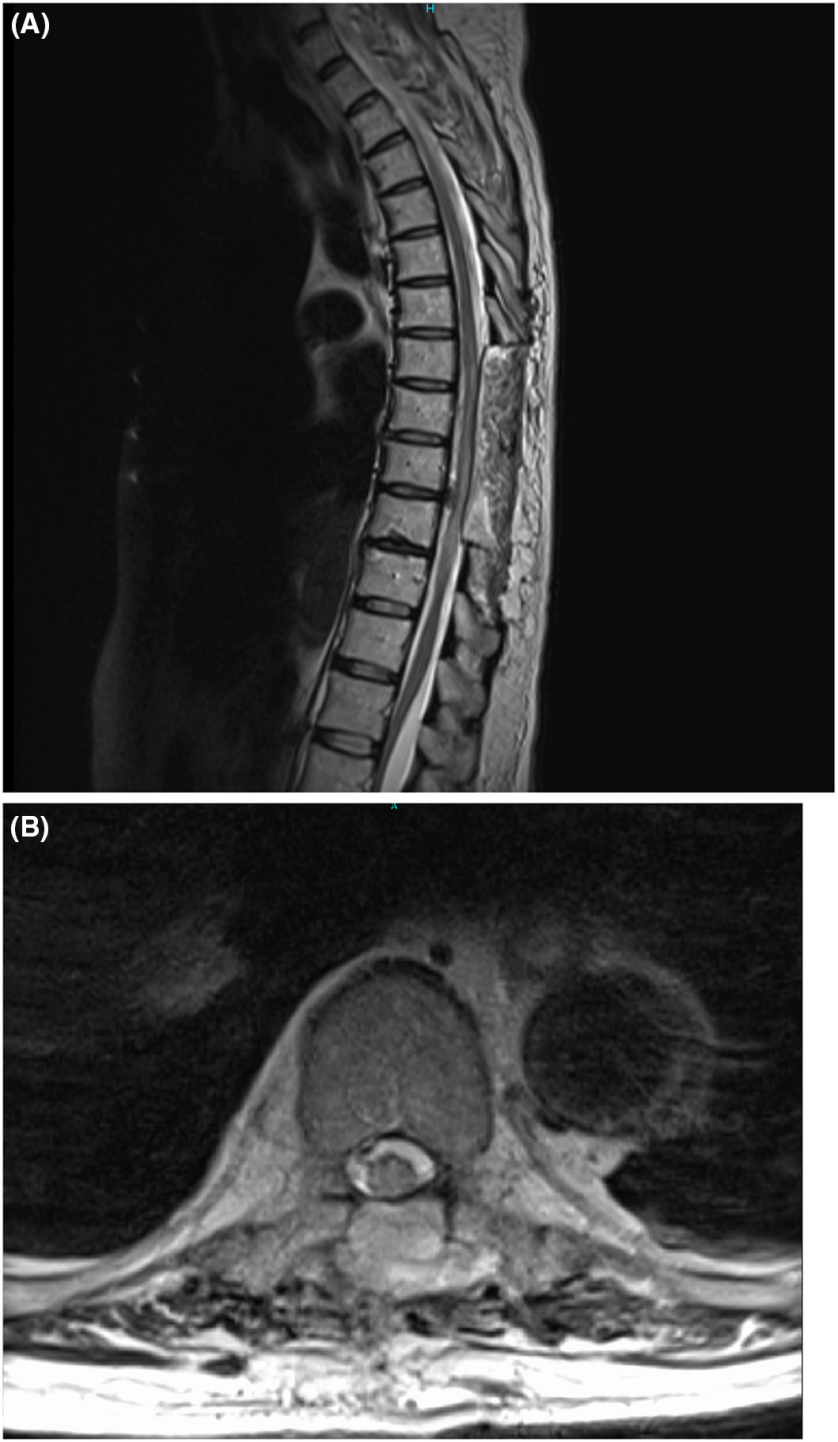
MRI 2 weeks after surgery. (A) Sagittal T2‐weighted MRI revealed that the compression of the spinal cord due to hematoma was relieved from the T7 level to the T10 level. (B) Axial T2‐weighted MRI also showed that spinal cord compression at the T10 level was relieved.

**FIGURE 3:**
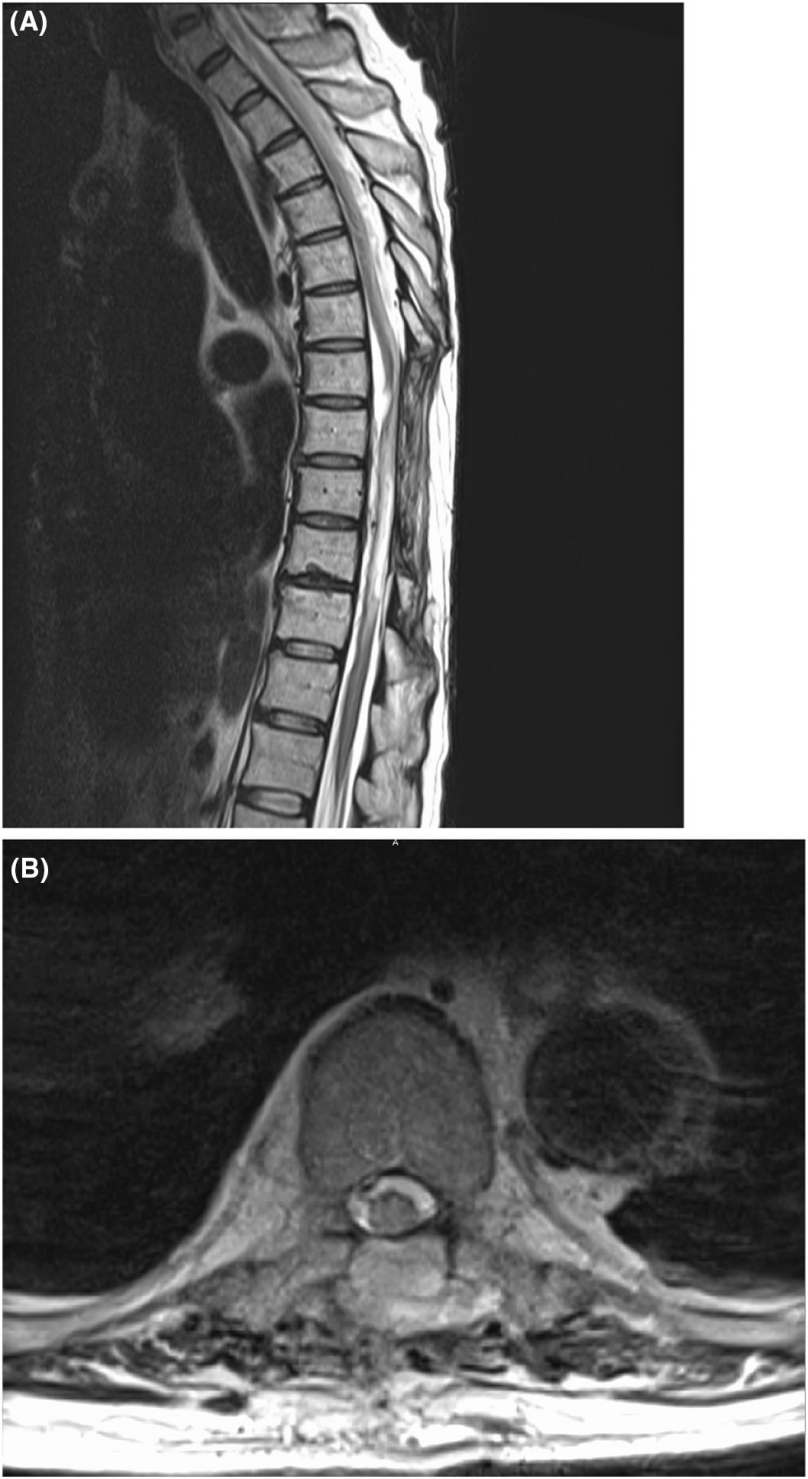
MRI 3 months after surgery. (A) Sagittal T2‐weighted MRI showed cerebrospinal fluid collecting on the ventral side of the spinal cord, with findings of adhesive arachnoiditis following subdural hematoma. (B) Axial T2‐weighted MRI also showed fluid collection on the ventral side.

The surgery was completed; however, the complete paralysis of both lower limbs and the bladder and rectal disturbances persisted. An MRI performed 2 weeks after surgery revealed that the compression of the spinal cord by the hematoma had been alleviated (Figure 2A, B). Despite the fact that we completed hemostasis, no improvement was observed in the symptoms of bilateral lower limb paralysis or the bladder and rectal disturbances. An MRI 3 months after surgery showed the presence of cerebrospinal fluid accumulating on the ventral side of the spinal cord, which is thought to be due to adhesive arachnoiditis following the SDH (Figure 3A,B). Adhesive arachnoiditis was observed, but there were no changes in neurological findings. At 4 years follow‐up, there was no noted improvement in the bilateral lower limb paralysis and the bladder and rectal functions.

## 3 DISCUSSION

### 3.1 Background and neurological symptoms of SSDH

Spinal subdural hematoma was a rare neurological emergency accounting for only 4.1% of all intraspinal hematomas.^8^ In 2020, Kosarchuk et al.^9^ reviewed SSDH in patients receiving anticoagulant therapy and found 24 case reports, with an equal gender ratio (12 males and 12 females) and a mean age of 63.0 years (range 38–80). Atrial fibrillation was the most common condition (15 patients), followed by cardiovascular disease (three patients), stroke (two patients), venous thromboembolism (two patients), heart valve replacement (two patients), and others (one patient). Warfarin use was most common in this series of SSDH patients with 42% (10/24). This patient was also taking warfarin and aspirin. As newer direct oral anticoagulants (DOACs) have become more widespread, the incidence of SSDH with DOACs would also be an issue to investigate. Therefore, the administration of anticoagulants is a risk factor for the development of SSDH.

The clinical presentation of SSDH includes sudden‐onset back pain followed by spinal cord disorders such as motor, sensory, and bladder‐rectal disturbances due to spinal cord compression.^10^ The severity of disability in SSDH has been reported to vary enormously, from pain alone without motor or sensory impairment to complete quadriplegia.^1^, ^3^, ^5^ However, many cases seem to have symptoms of low back pain and paraplegia similar to this case.^3^, ^5^

### 3.2 Characteristic imaging findings of SSDH

A potential pathogenic mechanism for SSDH has been proposed as a rupture of the vasculature within the subarachnoid or subdural space.^11^, ^12^ The localization of the hematoma is crucial for diagnosis and subsequent surgery. Differentiation from epidural hematoma is important for surgical planning, including the presence or absence of a dural incision.

For imaging diagnosis, CT and MRI are used. Since the dura mater is firmly adhered to the posterior longitudinal ligament, epidural hemorrhages often occur on the dorsal side, while subdural hemorrhages often occur on the ventral side.^8^, ^13^ In plain CT, the hematoma is hyperintense and the fat is hypointense, making it relatively easy to delineate the epidural fat region with clear contrast, which is useful for localization diagnosis.^14^

MRI is the gold standard of choice for diagnosis.

In MRI sagittal images, epidural hematomas often appear as biconvex hematomas with tapered upper and lower edges.^13^ On the other hand, subdural hemorrhage occurs inside the dura mater and often appears in a crescent shape.^18^ Although this case was not a crescent shape, a SDH was suspected based on the presence of a hematoma in the dura mater.

### 3.3 Management of SSDH

The management strategy for SSDH remains controversial. Patients with mild or moderate symptoms can be treated conservatively, while those with severe symptoms require urgent surgical decompression and thrombectomy to prevent neurological sequelae and death.^1^, ^3^, ^9^ Of the 24 cases of SSDH occurring during anticoagulant therapy by Kosarchuk et al.^9^, ^12^ 16 cases underwent surgery and 8 were treated conservatively; five did not improve in prognosis, 13 partially improved, five recovered completely and one died of cardiac arrest. Among SSDH patients whose general condition was stable, those with decreased leg muscle strength or bladder and rectal disorders often underwent surgical treatment or drainage tube placement.^13^ As for the case that died, conservative treatment was chosen because it was complicated by cranial subarachnoid hemorrhage.^18^ Four out of five patients who made a complete recovery were treated conservatively, and one underwent surgical treatment.^9^ Rettenmaier et al.^3^ reported 12 patients with complete paraplegia, eight underwent surgical intervention, and the remaining 4 were managed conservatively. Outcomes for patients who underwent surgical decompression included partial recovery (3/8), poor recovery, or no recovery (3/8).^3^ Additionally, patients with complete paraplegia who were managed conservatively experienced complete or good recovery (50%; 2/4), or partial recovery (50%; 2/4).^3^ The remaining two cases died.

Some studies in the literature suggest that conservative management was associated with better outcomes, which may be related to a bias whereby more disabled patients are selected for surgical intervention, whereas less symptomatic patients are selected for medical management.^1^, ^16^ Patients with mild or moderate symptoms can be treated conservatively, while those with severe symptoms require urgent surgical decompression and thrombectomy to prevent neurological sequelae and death.^1^, ^3^, ^9^

According to a review by Pereira et al., important prognostic factors include neurological status at presentation, the presence of coagulation abnormalities, lumbar puncture status, and associated diseases.^3^ They found that for hematoma progression, the presence of surgery or SAH were not significant predictors of prognosis. In addition, Pereira et al.^1^ reported that the neurological severity at the time of onset is one of the factors influencing the outcome of nontraumatic spontaneous acute SSDHs. In fact, in this case, the patient had severe neurological deficits with complete paralysis of both lower limbs and the bladder and rectal disturbances at presentation, surgery was performed 13 h after onset, but the patient did not recover, so neurological deterioration may be important.

Ohayon et al.^15^ also performed surgical treatment for nontraumatic spontaneous SSDH and reported improvement in bilateral lower limb paresis and bladder‐rectal dysfunction. Dezawa et al.^17^ reported that bilateral lower limb paralysis and bladder‐rectal dysfunction improved after surgical treatment of nontraumatic spontaneous SSDH within 14 h.

Both good and poor postoperative outcomes have been reported after surgical removal of the hematoma, and although surgery does not always lead to a good recovery of symptoms, we believe that surgery is recommended if there is worsening of neurological findings, such as paralysis.

Currently, in the field of neurosurgery, hematoma removal using endoscopy is performed. According to Cutler et al,^18^ endoscopic drainage in chronic SDH has been reported to significantly lower the recurrence rate compared to conventional surgical drainage. In the field of spinal surgery, there are no reports of endoscopic drainage for SSDH, but it may be considered as a future treatment option.

In this case, findings of adhesive arachnoiditis were observed. There are scattered reports that clinical findings and MRI manifestations of adhesive arachnoiditis do not always correlate.^19^ However, adhesive arachnoiditis can sometimes cause irreversible bilateral lower limb paralysis and must be considered in the differential diagnosis.^20^

### 3.4 Reminder to clinicians

With the advent of a globally aging society, the intake of anticoagulants for thrombosis prevention has increased. Therefore, it is expected that the incidence of SSDH will continue to rise. SSDH is an emergency condition, and early diagnosis is very important. Early diagnosis is essential for choosing the appropriate surgical treatment and can significantly affect the prognosis.

In the future, more attention should be paid to SSDH associated with antithrombotic therapy. To do this, a thorough clinical evaluation is necessary to consider lesions at the thoraco‐lumbar spine level and recognize characteristic imaging findings, which will enable accurate diagnosis and determination of the appropriate treatment plan.

We believe that the patient should be transported to a medical institution where a spine surgeon can provide treatment as soon as possible.

## 4 CONCLUSION

We report on a rare case of SSDH we experienced. The intake of anticoagulants increases the risk of SSDH. In this case, even though early surgical intervention was performed after the onset of SSDH, there was no improvement in the bilateral lower limb paralysis and bladder and rectal disorders. However, the literature shows cases that have recovered, indicating that early detection and surgical intervention are very important for the prognosis of SSDH.

## AUTHOR CONTRIBUTIONS

**Hayato Ito:** Writing – original draft; writing – review and editing. **Hirohito Hirata:** Writing – review and editing. **Tomohito Yoshihara:** Writing – review and editing. **Masatsugu Tsukamoto:** Writing – review and editing. **Kazuyuki Watanabe:** Writing – review and editing. **Yoshiaki Egashira:** Writing – review and editing. **Masaaki Mawatari:** Supervision. **Tadatsugu Morimoto:** Supervision; writing – review and editing.

## FUNDING INFORMATION

None.

## CONFLICT OF INTEREST STATEMENT

The authors declare that they have no competing interests.

## ETHICS STATEMENT

Not mandated for case reports.

## CONSENT

Written informed consent was obtained from the patient to publish this report in accordance with the journal's patient consent policy.

## Data Availability

Data pertaining to this case can be obtained by contacting the corresponding author.
